# T-Cell Receptor Repertoire Analysis with Computational Tools—An Immunologist’s Perspective

**DOI:** 10.3390/cells10123582

**Published:** 2021-12-18

**Authors:** Mahima Arunkumar, Christina E. Zielinski

**Affiliations:** 1 Department of Infection Immunology, Leibniz Institute for Natural Product Research and Infection Biology, Hans-Knoell-Institute, 07745 Jena, Germany; M.Arunkumar@campus.lmu.de; 2 Department of Biological Sciences, Friedrich Schiller University, 07743 Jena, Germany; 3 Bioinformatics, Ludwig Maximilians University Munich, 80539 Munich, Germany

**Keywords:** T-cell receptor repertoire, bioinformatic analysis, T cells, systems immunology

## Abstract

Over the last few years, there has been a rapid expansion in the application of information technology to biological data. Particularly the field of immunology has seen great strides in recent years. The development of next-generation sequencing (NGS) and single-cell technologies also brought forth a revolution in the characterization of immune repertoires. T-cell receptor (TCR) repertoires carry comprehensive information on the history of an individual’s antigen exposure. They serve as correlates of host protection and tolerance, as well as biomarkers of immunological perturbation by natural infections, vaccines or immunotherapies. Their interrogation yields large amounts of data. This requires a suite of highly sophisticated bioinformatics tools to leverage the meaning and complexity of the large datasets. Many different tools and methods, specifically designed for various aspects of immunological research, have recently emerged. Thus, researchers are now confronted with the issue of having to choose the right kind of approach to analyze, visualize and ultimately solve their task at hand. In order to help immunologists to choose from the vastness of available tools for their data analysis, this review addresses and compares commonly used bioinformatics tools for TCR repertoire analysis and illustrates the advantages and limitations of these tools from an immunologist’s perspective.

## 1. Introduction

Long-lasting T- and B-cell responses are the hallmark of immunological memory. Quantitative shifts in T cells with distinct T-cell receptors occur as a result of proliferation and thus clonal expansion in response to cognate antigens, which can originate from a plethora of microbial pathogens but also autoantigens. Clonally expanded T cells express the same unique TCR. Some of them will persist after a contraction phase to provide long-term immunological memory, i.e., against viral antigens. They can therefore serve as biomarkers of host protection. All individual TCRs of a given individual comprise the TCR repertoire. It represents a reflection of the individual’s history of antigen exposures [1]. Correlation of antigen-specific T cells with their respective functional polarization and differentiation state provides further qualitative measures of protection and tolerance [1].

Despite many ingenious methods, technical limitations have made it difficult for many years to create a comprehensive overview of TCR repertoires, until highly specific methods based on next-generation sequencing were developed. They facilitated the parallel analysis of millions of TCR and BCR (B cell receptor) sequences. In particular, single-cell sequencing made it possible to determine the relationship and dynamics of antibody and TCR repertoires with more accuracy on the single-cell level. Thus, high-throughput sequencing has enabled the profiling of TCRs and BCRs in single cells and of transcriptomes at an impeccable resolution, which is greatly adding to our understanding of adaptive immune responses in health and disease [1,2,3].

The effectiveness of the adaptive immune system strongly depends on the diversity and availability of antigen receptors [4]. How does the adaptive immune system generate antigen receptors with such a huge diversity to cover recognition of a plethora of antigens? The answer is that complete genes that encode a variable region are generated by the somatic recombination of separate gene segments. Multiple contiguous variable gene segments (TCRV) are present at each immune globulin locus. The variable (V), diversity (D) and joining (J) segments are rearranged in a stochastic fashion, which is the cause for the combinatorial diversity in antigen receptors encoding the complementary-determining region three (CDR3) of the TCRb receptor [5]. The hypothetical diversity of the TCR repertoire achieved by combinatorial diversity is by itself huge, with the actual heterogeneity increasing even more by the process of non-template insertion and deletion, as well as pairing of heterogeneous chains. It probably spans the range of 10^15^–10^20^, thus exceeding the number of 10^12^ T cells that continuously patrol the body [1,4,6]. The massive hypothetical diversity of the TCR repertoire therefore results in only a limited number of T cells with a unique TCR within an individual. Consequently, rare T-cell clones can easily be overlooked. Novel sequencing-based technologies have strongly improved this bottleneck [2,7].

**Figure 1:**
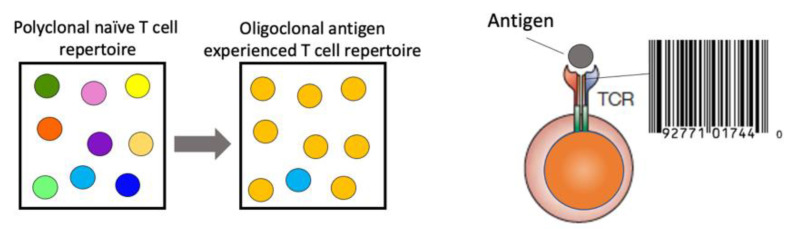
Clonal outgrowth of a T cell with a unique TCR (barcode) due to stimulation with its cognate antigen that generates shifts in the composition of TCR repertoire.

The information gained from a comprehensive TCR repertoire analysis is immense. The TCR represents a unique identification of T-cell clones due to the low probability of somatic recombination of the exact V(D)J rearrangement in a second T cell within the same individual. An increase in specific TCR frequency can act as a proxy for antigen-specific immune responses, resulting in clonal expansion of antigen-specific T cells with the same TCR (Figure 1). Longitudinal analysis of the TCR repertoire, paired with functional assessment of the T-cell quality, can yield insights into the ensuing immune response to the respective antigen, i.e., a pathogen. This is essential to gain an understanding for the generation of protective immunity, the pathogenesis of immune-mediated diseases and for the design of therapeutic strategies.

How can we tackle the magnitude and diversity of the human TCR repertoire to map the history of antigen encounter and to retrieve immunological correlates of host protection and tolerance? In the following sections, we provide an overview of the currently available methodologies for repertoire analysis by looking at five specific tools that are frequently used by immunologists. In addition, we describe the different aspects, including the advantages and disadvantages, that the immunologist should consider when choosing the appropriate method for a given research question.

## 2. Data Analysis for Exploring Immune Repertoires

**Figure 2:**
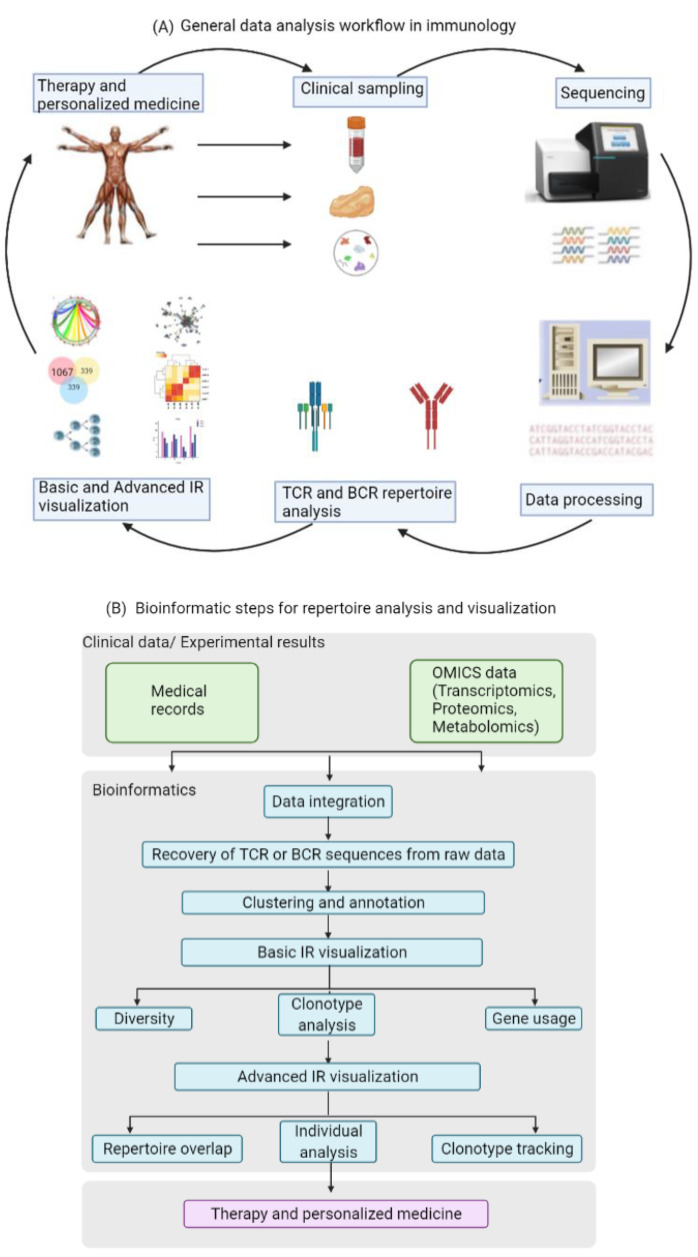
Standardized data analysis workflow for TCR/BCR repertoire analysis. (**A**) Pipeline from the clinic to the sequencing results. (**B**) Bioinformatic steps for basic and advanced immune repertoire analysis and visualization.

A variety of computational and statistical methods are currently available for studying large sets of raw data [7,8,9,10,11,12,13]. Most of these tools are helpful for identifying features and patterns in the data that have some functional or biochemical significance. The first step in the analysis is the recovery of TCR or BCR sequences from the raw data, and this step is then followed by clustering and annotation. The next step in the workflow is the visualization of the immune repertoire (IR), which commonly includes clonotype abundance, diversity and V(D)J usage. Especially the calculation of gene usage in the different samples is of significance, since a change in the usage of specific genes may be due to alterations in the repertoire caused by the respective underlying disease or immunological perturbation. The last step in the data analysis of the immune repertoire traditionally involves the visualization of repertoire overlap and clustering, as well as specialized analysis of individual clonotypes and generation of publication-ready figures. Particularly changes in TCR overlap and diversity over time are important measurements that could be informative of disease progression. Figure 2 illustrates the data and information flow in standard immunology research.

To determine TCR diversity, several indices can be employed. In T-cell repertoires, diversity takes the clonal composition into account, specifically the number of unique TCR sequences (richness) and the relative abundance of these sequences (evenness). However, it is important to point out that it is not possible to directly measure the total TCR diversity in a given sample. Since the TCR repertoire is highly diverse and the distributions of individual TCRs can be heavily skewed, the diversity cannot be evaluated from experimental samples alone [4,6,14,15].

Most of the diversity indices stem from the information theory to quantify ecosystems biodiversity. Commonly used indices are the Shannon index, the Inverse Simpson index, Gini coefficient or the DE50 score [4,15,16,17,18].

The Shannon index accounts for sample richness and evenness. A large Shannon index value implies that the distribution of the CDR3 sequences is more diverse. When using the Shannon index to compare different samples with each other, researchers must be careful, as this index assumes that the distributions of the clonal frequencies of the samples are similar to each other, and this may not necessarily be true for every sample being compared. Moreover, the Shannon index is sensitive to low-frequency reads. Thus, variations and changes in low-frequency reads could affect the outcome of this diversity index [15,16].

The Inverse Simpson index, in contrast to the Shannon index, emphasizes high-frequency reads, and therefore researchers should only use the Inverse Simpson index when their dataset contains high-frequency reads. High values of this index indicate an even distribution of TCR clones, and low values indicate enrichment of T-cell clones [15,16].

The Gini coefficient measures the inequality among values of a frequency distribution. It was originally proposed to measure the inequality of income or wealth across countries but can also be used in immunology. The scale ranges between zero and one. Zero stands for total equality of clones; thus, all clones will have identical frequencies. On the other hand, one stands for total inequality, thus indicating sample oligoclonality. This index can be used to gauge the inequality of the frequency distribution between different clonotypes in each sample and give a summary of the clonotype abundance distribution [19,20,21,22,23].

The DE50 (diversity evenness score) is considered an indicator for the degree of clonality in a given dataset. Summing up the number of reads in ranked order, from high to low, DE50 presents how many unique reads can be found within 50% of in-frame reads. A low value indicates a high clonality level [14,15,16,17,18]. Since each index only takes a fraction of different diversity aspects into account, they all have certain biases when applied to various underlying populations in terms of size and abundance distribution. If two repertoires are being compared, it is possible that one diversity measure shows a certain trend, while the other shows a very different result (or even the opposite result), considering that they represent different aspects of the underlying abundance distributions.

Regarding overlap measures, i.e., a measure on how similar or different two sets of data are, the Morisita–Horn index and the Jaccard index are commonly used in immunology [14,16]. In contrast to the α-diversity measures (e.g., the Shannon index) discussed above, these overlapping measures are often termed as β-diversity measures [18].

The Morisita–Horn index is a statistical measure of dispersion of individuals in a population. It accounts for both the number and abundance of shared TCRs between two repertoires, and its score ranges between 0, meaning no overlap, and 1, meaning all clones overlap at similar frequencies. The Jaccard index measures similarity between sample sets and is defined as the size of the intersection divided by the size of the union of the sample sets. It also ranges between 1 and 0, where 1 indicates complete overlap and 0 represents no overlap. The Yu–Clayton index is one of the few similarity indices that can detect and compare the presence and abundance of same TCRs among samples [22]. It has, however, only rarely been used so far in the field of immunology, as compared to the Morisita–Horn index or the Jaccard index. Most of these indices are very similar (especially the Morisita–Horn index and the Jaccard index), but they differ in the consideration they give to factors such as the species richness or the evenness of the given data. Therefore, it is very important for scientists to be watchful when comparing results obtained from different tools that use different techniques. Moreover, experimental sampling only partially estimates the overlap and diversity of repertoires [4,6,15]. Therefore, researchers must be careful when dealing with immune repertoire data as uniformity between samples is important. To this end, down-sampling or re-sampling are one of the commonly used strategies to generate more comparable data. Down-sampling refers to the reduction of the dataset to a more manageable size and thus working with a random sample without replacement from our original data. Re-sampling means that random data can be drawn with replacement from our original dataset in such a way that this is comparable to the original data. In this way, re-sampling allows us to make unbiased estimates, as it is drawn from unbiased samples.

## 3. Scirpy

Scirpy is a Python-toolkit that is used to analyze TCR repertoires from single-cell RNA sequencing (scRNA-seq) data [24,25]. It can easily be integrated with the Scanpy library, which is a toolkit for analyzing single-cell gene expression data. Scanpy is one of the standard tools for preprocessing, visualization, clustering, pseudotime trajectory inference, differential expression analysis and simulation of gene regulatory networks [26,27].

Scirpy is a pipeline and is available for the characterization of T-cell receptors. It can be used for the visualization of immune repertoires from single cells and for integration with transcriptomic data to characterize the TCRs of single T cells. Starting with the process of data loading, Scirpy supports a variety of data formats, including 10× Genomics CellRanger, TraCeR, BraCer or AIRR-compliant data [20,27,28]. Detailed tutorials with examples on data loading and core analysis make it easy for less experienced researchers to work with Scirpy.

Scirpy enables the investigation of the composition and phenotypes of both single and dual TCRs in T cells. With Scirpy, researchers can inspect TCR chain configurations and explore the abundance, diversity, expansion and overlap of clonotype repertoires across samples, patients or cell clusters. It can also integrate transcriptomics data. Finally, it is possible to investigate the intricate relationship between cells and clonotypes, as well as analyze the distribution of CDR3 sequence lengths and V(D)J gene usages. Regarding clonotype analysis, Scirpy implements a network-based approach that enables clustering of cells into clonotypes based on having either identical CDR3 nucleotide sequences, identical CDR3 amino acid sequences or similar CDR3 amino acid sequences based on pairwise sequence alignment. The sequence-alignment-based networks offer the opportunity to identify cells that might recognize the same epitopes.

**Figure 3:**
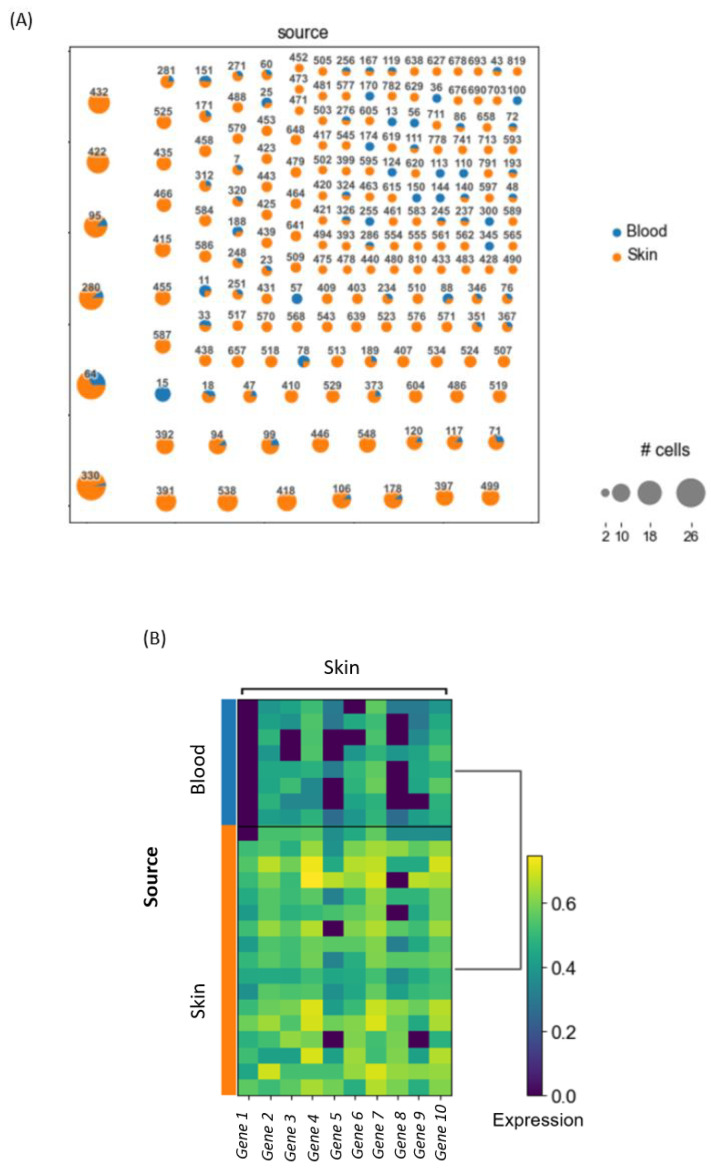
(**A**) Example of a clonotype network constructed by Scirpy, using the default parameters in order to showcase all clones found in the dataset comprising T cells from matched skin and blood of a given patient. (**B**) Heatmap showing the top 10 differentially expressed genes for the shared clone with the clone ID 64 (as depicted in (**A**)), using default parameters in Scirpy.

Let us assume that a basic clonotype analysis on a 10× dataset, which contains blood and skin samples from a patient, should be conducted. After loading, preprocessing and normalizing the data, all T cells, based on the criteria of having a TCR, will be extracted from the dataset. Then the clonality can be assessed and a clonotype network can be constructed by showing all the clones in the dataset based on their assigned clone ID. This allows for the identification of shared clones, i.e., clones that can be found in blood, as well as skin samples. In these shared clones, differential gene expression between blood and skin within each shared clone can be analyzed. This identifies whether T cells with identical precursors differ in gene expression as a result of their differential location (skin versus blood). Based on the top differentially expressed genes, we can formulate a certain hypothesis about our data that can then be accepted or rejected depending on further downstream analysis. An example of the results is visualized in Figure 3.

All results also have the advantage of being generated in a publication-ready style. It can therefore be concluded that Scirpy provides a big range of methods for analyzing T-cell repertoires from single-cell sequencing data. It is open-source and has detailed tutorials to support researchers who conduct the analysis. In this way, prior in-depth knowledge in Python is certainly useful, but not necessary. On the other hand, Scirpy does have two main shortcomings, namely that it does not support bulk data formats or BCR-related analysis. Thus, if this kind of analysis needs to be conducted, researchers need to refer to other tools.

## 4. Immunarch

Another open-source tool (previously known as tcR) is an R package called Immunarch [29]. It is fully interoperable with Seurat. Seurat, also available as a Bioconductor package, is an R package designed for quality control, analysis and exploration of single-cell RNA-seq data, and thus it contains implementations of commonly applied techniques for exploring single-cell expression data [30]. Immunarch offers data loading, analysis and visualization for all popular TCR and BCR analysis and post-analysis formats, including single-cell data: ImmunoSEQ, IMGT, MiTCR, MiXCR, MiGEC, MigMap, VDJtools, Immunarch, AIRR, 10× Genomics and ArcherDX [27,28,29,31,32,33,34,35]. Since this tool is constantly being updated, even more useful methods and applications are expected to be available in the future [29]. Additionally, Immunarch works on almost any kind of data, from R data frames and data tables to databases such as MonetDB or Apache Spark data frames via sparklyr. Not only does Immunarch accept all standard immunosequencing formats, but it also automatically detects and parses the format of the uploaded data.

Immunarch implements most of the commonly used analysis methods, such as clonality analysis; estimation of repertoire similarities in the distribution of clonotypes; gene usage and kmer distribution measures; repertoire diversity analysis; clonotype tracking between samples and across time points; and annotation of clonotypes, using external immune receptor databases. This includes the frequently used databases: VDJDB, McPAS-TCR and TBAdb from PIRD [32,33,34]. All results are generated in a publication style. However, Immunarch also has a built-in tool for additional visualization manipulation, such as changing font sizes, text angles, titles or legends.

Immunarch is beginner-friendly, because a researcher requires little to no knowledge in R in order to conduct the analysis, and most methods are incorporated into a couple of main functions with simple naming. Moreover, Immunarch includes a comprehensive tutorial with examples for an easy start. All of these features make the Immunarch package very popular for research, especially when TCR analysis and clonotype comparison need to be conducted. For example, recently the Immunarch package was used for BCR analysis, which demonstrated that a single human V_H_-gene enables a broad antibody response in the blood that targets bacterial lipopolysaccharides [36].

In sum, Immunarch provides a vast range of methods for analyzing both T-cell and B-cell repertoires. The fact that it is an open-source tool and includes detailed explanations and tutorials makes it easy for beginners to conduct advanced analysis in a fast and efficient manner. The only main drawback is the fact that it currently provides paired chain information only for 10× Genomics data.

## 5. ImmunoSEQ Analyzer 3.0

ImmunoSEQ Analyzer 3.0 is an online web tool for data exploration provided by the company Adaptive Biotechnologies [35]. It enables researchers to understand the contours and dimensions of their data. The immunoSEQ analyzer 3.0 offers all the basic analysis and visualization methods for immunological research. The Sample Overview dashboard includes information regarding the number of productive templates, rearrangements, maximal productive frequencies and clonalities of each sample. The analyzer supports various views, which make it possible to see the details of single samples or even compare two or more samples with each other. The different views allow researchers to identify complete sequence information for all unique TCR or BCR rearrangements in a sample, track how particular clones expand across samples or tissues and compare the gene usage between samples. Additionally, pairwise scatterplots can be generated that reveal the relative abundance of every detected clone. Moreover, the analyzer features tools to conduct additional statistical tests and metrics for immunosequencing data. This includes a tool for plotting a Venn diagram, a down-sampling tool for comparing samples of different sizes, a differential abundance tool for comparing different samples and additional diversity metrics. The generated results are publication-ready and can be exported easily, along with the sample data.

The main advantage of using the analyzer is that it contains a massive database of TCR and BCR sequences. Researchers can incorporate millions of sequences of public data and control samples by using the immunoSEQ Analyzer, compare analysis from multiple investigators, organize all samples into experiment-specific folders and share the created projects with colleagues. The data can even be shared with off-site collaborators by making use of the option of showcasing the data in the company’s immuneACCESS database. The biological and translational applications for this methodology are manifold. For example, the immunological correlates of vaccines against SARS-CoV-2 have recently been interrogated with this methodology. Researchers found that polyfunctional spike protein specific Th1 corresponds to a diverse TCR repertoire [33]. In addition, immune cell lineage tracing and cellular developmental pathways can be investigated. It has been shown, for example, that tissue resident memory T cells (T_RM_) and central memory T cells (T_CM_) share identical naïve precursor cells due to their overlapping clonal origin [35].

However, this tool also has its drawbacks, such as the fact that the immunoSEQ Analyzer is specifically designed around immunoSEQ data and does not support outside upload. This implies that analysis with non-immunoSEQ data needs to be carried out by using other methods. Additionally, chain pairing information is not supported at present. It is also necessary to mention that it is not open-source software but a commercial tool. However, researchers can make use of the option of creating a free account first in order to explore the full range of the immunoSEQ Analyzer 3.0, including all integrated tools.

## 6. Immcantation Framework

The Immcantation framework is a massive and powerful analytical system for high-throughput AIRR-seq datasets. Starting with raw reads, this tool contains several Python and R packages that can conduct preprocessing and repertoire analysis and determine population structures. There are eleven core and contributed packages. The seven core packages are pRESTO, Change-O, Alakazam, SHazaM, TIgGER, SCOPer and prestoR. The four contributed packages are [37,38] RDI, RAbHIT, IgPhyML and sumrep [39,40,41]. All plots are generated in a publication-ready style.

Starting with the tool pRESTO, researchers can perform preprocessing, from raw sequences to paired-end assembly. The main aim of preprocessing is to transform raw reads into sequences where errors have been eliminated. Overall, pRESTO supports multiplexed and RACE samples. It can also perform de-multiplexing, which is usually already performed in sequencing facilities. Moreover, pRESTO supports single-end sequencing, too. Read processing can be performed with or without UMI inclusion, making it compatible for various protocols a researcher might use [3,8]. After this, the pRESTO report package (prestoR) can be used to generate plots.

Regarding clonotyping, the Immcantation framework provides a package called Change-O for standardizing the output of the V(D)J reference alignment software, such as IMGT or IgBLAST [42,43]; clonal clustering; and germline reconstruction. Change-O allows for the processing of reads that contain a premature stop codon. Researchers can also group clonotypes by J or V allele and other parameters, such as the nucleotide Hamming distance, amino acid Hamming distance and many others. When performing hierarchical clustering, researchers are given the choice between single, average or complete linkage [37].

The included R package Alakazam can plot a repertoire lineage tree by using the clone outputs from Change-O. Various diversity measures, such as the species richness, the Shannon index or the inverse Simpson index, are also performed by Alakazam. Additionally, Alakazam calculates V(D)J alleles, determines gene usage, infers clonal abundance and lists the chemical properties of the amino acid sequences [37]. It also provides the option to generate rarefaction curves.

This suite of packages, all integrated into one big framework, makes the Immcantation Portal a mighty asset for almost any kind of repertoire analysis a researcher might want to conduct in the realm of immunology. An example is given by a recent publication that demonstrated stratification of celiac disease patients and controls by naïve B-cell repertoires by using the TIgGER package to conduct preliminary data processing in order to deduce new alleles and a personalized genotype for all individuals in their data [37,38].

In sum, the Immcantation framework contains various packages which are highly valuable for immunological research. Immcantation includes pRESTO, which handles all stages of sequence processing from raw reads up to V(D)J gene assignment. To facilitate advanced repertoire analysis, Immcantation also contains methods for novel V gene allele detection (TIgGER), subject-specific germline genotype identification, B-cell clone assignment (Change-O and SCOPer), lineage tree construction and analysis (IgPhyML), somatic mutation profiling and selection analysis (BASELINe) [40,41]. Immcantation can start from raw data or read the output of common V(D)J assignment tools, such as IgBLAST. It also supports MiAIRR and the AIRR Community data standard and includes tools to facilitate MiAIRR-compliant submissions to NCBI repositories [28].

However, the Immcantation framework, being a mixture of many sub-packages, may seem confusing and overwhelming at first, even though each package is thoroughly documented. Moreover, Immcantation offers various summary functions for AIRR-seq data, but it does not have a sophisticated method for comparing and visualizing these summaries. Many summaries of interest are implemented in one of the many sub-packages, but there is no single standard data format. This can be a cause for struggle when comparing summaries across packages [37,38,40,41].

## 7. VDJtools

VDJtools is an open-source software framework for TCR and BCR analysis and is based on Java [44,45]. It can analyze the output of the following VDJ junction mapping and analysis platforms: MiTCR, MiGEC, IgBlast, IMGT, ImmunoSEQ, VDJdb, Vidjil, RTCR, MiXCR and ImSEQ.

The framework also has a built-in tool, called *correct*, that performs frequency-based correction to eliminate erroneous clonotypes. Additionally, VDJtools provides a variety of other filtering options, such as filtering non-functional clonotypes, filtering out all clonotypes found in another sample, filtering by frequency and filtering V(D)J segments that match a specified segment set. VDJtools allows for basic, as well as advanced, IR visualization by applying a diverse set of methods and strategies. Researchers can make use of the command line tool, which is user-friendly and well documented, so that immunologists with little computational background can easily generate publication-ready plots or simply make use of the tabular outputs. For each sample, VDJtools calculates basic statistics of read counts, mean clonotype size and number of non-functional clonotypes. It determines VJ gene usage and the distribution of clonotype abundance by CDR3 sequence lengths [45].

As for diversity indexes, the framework provides many methods, such as Chao 1, Efron–Thisted, Shannon–Wiener index, Normalized Shannon–Wiener index and Inverse Simpson index. The framework performs a comprehensive analysis of clonotype sharing and clonotype tracking, as well. Data can be visualized as scatter plots of overlapping clonotype abundance, abundance plots, sequence clustering dendrograms and (pairwise) overlap plots. VDJtools includes a built-in tool called CalcPairwiseDistances, which performs a pairwise overlap for a set of samples and computes a list of repertoire similarity measures. Additionally, by using databases, such as VDJdb, that contain CDR3 sequences, variable and joining segments can be obtained. VDJtools can also annotate samples based on VDJ junction matching [45].

Considering all features, it becomes evident that VDJtools can be beneficial to researchers for general TCR and BCR data analysis. An example can be found in a recent publication that used the VDJtools software to analyze and visualize their MiXCR bulk TCR output data to demonstrate that a conserved TCR signature dominates a highly polyclonal T-cell expansion during the acute phase of a malaria infection [46,47]. The following publication is another example where VDJtools was used on MiXCR data in order to calculate their average TCR repertoire characteristics weighted by clonotype size. The results indicated that memory CD4^+^ T cells are also generated in the human fetal intestine, and this was an unexpected, considering that the fetus is thought to be protected from exposure to foreign antigens [48,49,50].

In sum, VDJtools provides basic, as well as advanced, methods for analyzing T-cell and B-cell repertoires supporting various file inputs. It is an open-source tool and includes a comprehensive documentation, making it easy to work with. However, VDJtools does have a few major shortcomings, namely that it does not provide support for 10× Genomics or Smart-seq2 data yet. Moreover, paired chain information is not included. Thus, if this kind of analysis needs to be conducted, researchers are advised to use other tools.

## 8. Discussion

Other sophisticated tools worth mentioning are CoNGA (clonotype neighbor graph analysis) and scRepertoire. CoNGA is a graph-based approach which identifies correlations between gene-expression data and TCR sequences through statistical analysis of gene expression and TCR similarity graphs. It can be useful when studying the complex relationships between TCR sequences and T-cell phenotypes in large heterogeneous single-cell datasets [42,43,51,52,53,54,55]. The R-based tool scRepertoire is used for single-cell immune receptor analysis, and it combines mRNA and immune profiling for data derived from 10× Genomics Chromium Immune Profiling for TCR and BCR analysis [27,54]. At this point, we can conclude that several tools and methods for TCR and BCR repertoire analysis and clonotype identification exist. In this review article, we have illustrated the capabilities, advantages and disadvantages of five commonly used tools in immunology. Table 1 gives an overview of all the five tools that we employed in this review. However, there are many tools that are currently being used in immunology which have not been addressed in this review, and many newer tools are also emerging. This technological progress offers great opportunities for groundbreaking insights, and we can be sure that the methods and tools discussed here will continue to evolve and improve in the future. As of today, a gold-standard method for the field has not yet been identified. Depending on the purpose of the scientific study, some approaches may be more suitable than others. However, it is important to consciously select a method or a tool by keeping all strengths and weaknesses of each approach in mind. Finally, due to the possible method or tool-specific biases, scientists must always be very careful when comparing results obtained from different methods.

## Figures and Tables

**Table 1:** Overview of commonly used tools in immunology.

Tools	Data Format	Are TCR and BCR Analysis Possible?	Is It Open Source?	Are Costs Involved?	Are Detailed Tutorials Available?	Sharing and Collaborating on Data and Analysis Possible?
Scirpy	Only single cell data supports currently	BCR analysis not supported yet	Yes	No	Yes	No
Immunarch	Compatible with various data formats	TCR and BCR analysis possible	Yes	No	Yes	No
ImmunoSEQ analyzer 3.0	Does not directly support outside data upload	TCR and BCR analysis possible	No	Yes	Yes	Yes
Immcantation portal	Compatible with various data formats	TCR and BCR analysis possible	Yes	No	Yes	No
VDJtools	Compatible with various data formats	TCR and BCR analysis possible	Yes	No	Yes	No

## Data Availability

Not applicable.
